# Assessment of Acute Oral Toxicity of Thiolated Gum Ghatti in Rats

**DOI:** 10.3390/polym14183836

**Published:** 2022-09-14

**Authors:** Vivek Puri, Ameya Sharma, Pradeep Kumar, Kamal Dua, Kampanart Huanbutta, Inderbir Singh, Tanikan Sangnim

**Affiliations:** 1 Chitkara University School of Pharmacy, Chitkara University, Baddi 174103, Himachal Pradesh, India; 2 Department of Pharmacy and Pharmacology, School of Therapeutic Sciences, Faculty of Health Sciences, University of the Witwatersrand, Johannesburg 2000, South Africa; 3 Discipline of Pharmacy, Graduate School of Health, University of Technology Sydney, Ultimo, NSW 2007, Australia; 4 Faculty of Health, Australian Research Centre in Complementary and Integrative Medicine, University of Technology Sydney, Ultimo, NSW 2007, Australia; 5 Uttaranchal Institute of Pharmaceutical Sciences, Uttaranchal University, Dehradun 248007, Uttarakhand, India; 6 School of Pharmacy, Eastern Asia University, Thanyaburi 12110, Pathumthani, Thailand; 7 Chitkara College of Pharmacy, Chitkara University, Patiala 140401, Punjab, India; 8 Faculty of Pharmaceutical Sciences, Burapha University, Muang 20131, Chonburi, Thailand

**Keywords:** thiolated gum ghatti, oral toxicity study, blood clotting factor, Wistar rat

## Abstract

Various drug delivery systems were developed using a modified form of gum ghatti. Modifying gum ghatti using thioglycolic acid improves its mucoadhesive property, and hence, it is a suitable approach for the fabrication and development of controlled drug delivery systems. In accordance with regulatory guidelines, namely, the Organization for Economic Co-operation and Development’s (OECD) 423 guidelines, an acute oral dose toxicity study was performed to examine the toxicological effects of gum ghattiin an animal (Wistar rat) after a single oral dose administration of pure gum ghatti and thiolated gum ghatti. Orally administered pure and thiolated gum ghatti do not reveal any considerable change in the behavioral pattern, food intake, body weight, hematology, or clinical symptoms of treated animals. Furthermore, histopathological studies showed no pathological mutations in the vital organs of Wistar rats after the oral administration of single doses of both types of gumghatti (i.e., 300 mg/kg and 2000 mg/kg body weight). Whole blood clotting studies showed the low absorbance value of the modified gum (thiolated gum ghatti) in contrast to the pure gum and control, hence demonstrating its excellent clotting capability. The aforementioned toxicological study suggested that the oral administration of a single dose of pure and thiolated gum ghatti did not produce any toxicological effects in Wistar rats. Consequently, it could be a suitable and safe candidate for formulating various drug delivery systems.

## 1. Introduction

Gum ghatti (polysaccharide) is obtained from *Anogeissus Latifolia*, of the family Combretaceae, which is found in the deciduous forests of India and the Sri-Lankan sub-continent. Gum ghatti after hydrolysis produces sugars of a D-configuration, such as galactose, glucuronic acid, mannose, xylose, and one L-configuration such as arabinose and rhamnose (>1%) [1,2,3]. Naturally, it exists in a combination of varied salts including calcium, magnesium, potassium, and sodium. The polysaccharides present in gum ghatti are composed of 1 → 6 linked β-D-galactopyranose units with side chain residues of L-arabinofuranose, and they also have alternate units of 4-O-substituted and 2-O-substituted α-D-mannopyranose. Gum ghatti is used as a safe food additive in Japan [4], and it has been granted GRAS (Generally Recognized as Safe) status by the USFDA (United States Food and Drug Administration) (21 CFR 148.1333, FDA 2019) because of its emulsifying, stabilizing, and thickening properties. Gum ghatti is commercially used as a food additive and as a pharmaceutical excipient [5,6]. 

Thiolated polymers (Thiomers) are new class of mucoadhesive polymers that are used for developing a mucoadhesive formulation that will produce a sustained release. Thiolated gum ghatti could be obtained via the covalent attachment of a hydroxyl group of gum ghatti with a carboxyl group of thioglycolic acid. The thiolation procedure for Moringa gum [7], Gellan gum [8], Xanthan gum [9], Psyllium gum [10], Tamarind gum [11], Karaya gum [12], and gum ghatti [13] is already reported in the literature.

This research experiment focused upon assessing toxicity in Wistar rats after the oral administration of single doses of both pure gum and thiolated gum, with the aforementioned doses measuring at 300 mg/kg and 2000 mg/kg, respectively. The oral toxicity potential of pure and modified gum was examined using a variety of biochemical and clinical test parameters, as well as histopathological analysis. No evidence of genotoxicity and mutagenicity was reported in earlier studies [6]. Maroupt et al. performed oral toxicity studies for 90 days, using gum ghatti in rat models [14]. Kawahara et al. performed a four-week oral toxicity study. No toxicological or pathological evidence was reported at the high dose of 8000 mg/kg [15].

## 2. Materials and Methods

### 2.1. Chemicals

Gum ghatti was received as a gift sample from Hydrocolloid Plantations (New Delhi, India). Thioglycolic acid and all other chemicals and reagents were of analytical quality grade and were purchased from Loba Chemie Pvt. Ltd. (Mumbai, India). 

### 2.2. Animals

Wistar rats (weight; 200 g) were bought from the animal house of Lala Lajpat Rai University of Veterinary and Animal Sciences, Hisar, India. These animals were kept under observation in the central animal house of Chitkara University, Rajpura, Punjab, India, in order to conduct the current animal study. Under normal housing conditions, the animals were housed. All methods were carried out in accordance with the guidance set out by the Committee for the Purpose of Control and Supervision of Experiments on Animals (CPCSEA), the Government of India, and the Institutional Animal Ethics Committee (IAEC), the latter of which has reviewed and approved the research study protocol (IAEC/CCP/20/01/PR-004) [16]. We conducted all experimental protocols in compliance with the Animal Research: Reporting of In Vivo Experiments (ARRIVE) guidelines.

### 2.3. Synthesis and Characterization of Thiolated Gum Ghatti

**Figure 1:**
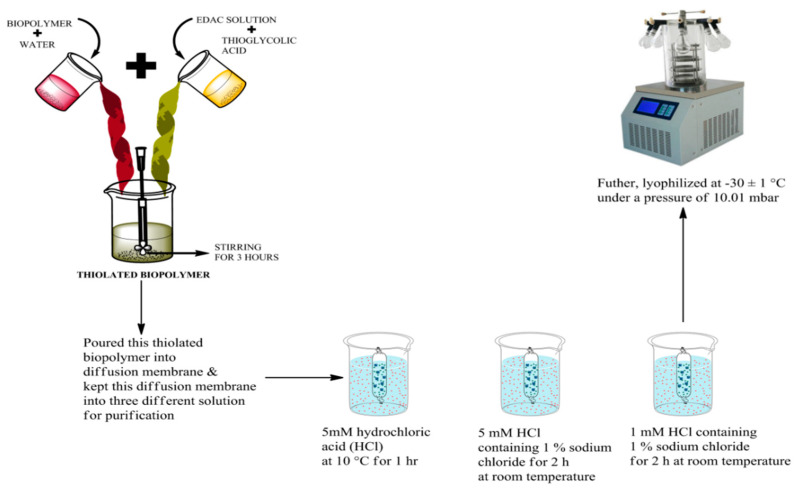
Diagrammatic representation of synthesis of thiolated gum ghatti using thioglycolic acid.

In 50 mL of deionized water, pure gum ghatti was dissolved; afterwards, a solution of 1-Ethyl-3-(3-Dimethylaminopropyl)carbodiimide (EDAC) and thioglycolic acid was added [13]. In addition, the prepared reaction mixture was poured into the dialysis membrane, and furthermore, it was placed once in 5 mM hydrochloric acid at 10 ± 1 °C; twice in a mixture of 5 mM HCl and 1% NaCl; and twice in a mixture of 1 mM HCl and 1% NaCl. The above dialyzed reaction mixture was lyophilized at −30 ± 1 °C (Allied frost, Delhi, India) and kept in a desiccator at a pressure of 10.01 mbar (Figure 1). Furthermore, the surface morphology and characteristic peaks, or presence of the functional groups of both of the gums, were examined using different techniques such as scanning electron microscopy (SEM) (Joel, fine coat ion sputter, JFC-1100), the Fourier transform infra-red (FTIR) spectrum (Alpha, Bruker, Japan), and X-ray diffraction analysis (XRD).The characterized results of SEM, FTIR, and XRD are depicted in the Supporting Information or Supplementary Files Figures S1–S3.

### 2.4. Experimental Procedure

**Figure 2:**
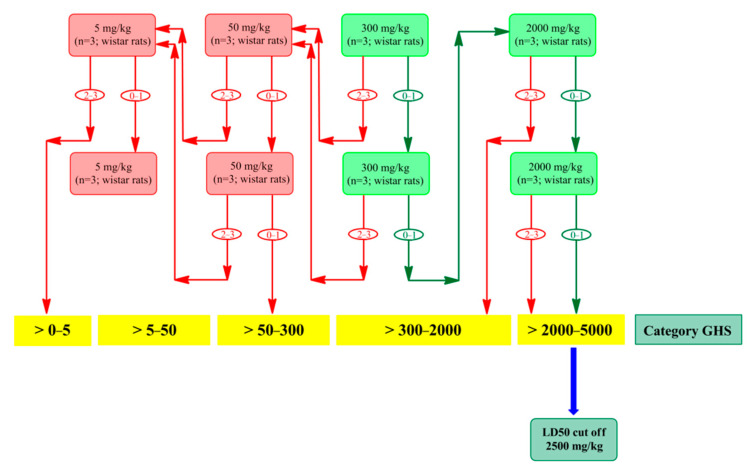
Treatment schedule procedure, as per the OECD’s 423 guidelines, with a starting dose of 300 mg/kg body weight; 0, 1, 2, 3 represents the number of moribund or dead animals at each step.

Both pure and thiolated gum ghatti were examined for their toxicity; in-vivo toxicological studies, in accordance with the OECD’s 423 guidelines [17,18], were followed for 14 days, and the acute single dose oral toxicity evaluation and detailed experimental schedule are shown in Figure 2 and Table 1. As per the OECD’s 423 guidelines, it is mandatory to handle three Wistar rats in each stage, and they also recommend using 300 mg/kg body weight (starting dose) if the substance to be tested is novel. A single oral dose of 300 mg/kg for both gums were prepared in water and administered to two different groups: Group-I (300 mg/kg body weight pure gum) and Group II (300 mg/kg body weight thiolated gum). In each group, there were three animals and stainless steel oral gavage feeding needles were used. After 4 h, no signs of toxicity, no behavioral changes in the animals, were observed after the administration of the dose; no significant changes were observed with respect to behavior and mortality, and no toxicity was produced. Hence, we increased the concentration in the single doses of both pure and thiolated gum ghatti (2000 mg/kg body weight), and the prepared doses were administered to two different groups, with three animals in each group: Group III (2000 mg/kg body weight pure gum) and Group IV (2000 mg/kg body weight thiolated gum), as per standard guidelines. An additional group with six animals was named Group V (control-0.9% *w*/*v* saline). Significant characteristics were observed on the last day of study, including biochemical, behavioral, and histological characteristics. All surviving rats were euthanized under mild anesthesia; afterwards, the rats’ cervixes were dislocated for the histopathological examination of various organs [19,20].

#### 2.4.1. Hematology and Biochemical Test Parameters

On last day of the treatment regime, blood samples of rats from each group were collected in EDTA coated vials from the retro orbital plexus of the rat for biochemical estimation and hematology evaluation. The Eurocount-TS Haematology Analyzer (Medsource Ozone Biomedicals Pvt. Ltd., Haryana, India) was employed for various hematological examinations, such as the determination ofhemoglobin concentration (HGB), the number of white blood cells (WBC), red blood cell count (RBC), platelet count (PLT), total leucocyte count (TLC), packed cell volume (PCV), differential leukocyte counts (DLC), the mean corpuscular hemoglobin concentration (MCHC), mean corpuscular hemoglobin (MCH), and mean corpuscular volume (MCV). Various liver function tests (for instance, tests that measure the total bilirubin, total protein, albumin, serum glutamic-oxaloacetic transaminase, serum glutamic-pyruvic transaminase, alkaline phosphatise, and total globulin content) and kidney function tests (for instance, tests that measureblood urea, serum creatinine, serum uric acid, serum potassium, serum sodium, serum calcium, and serum phosphorous content) were assessed by employing commercially available kits [21,22].

#### 2.4.2. Histopathology

On the 14th day of this study, the organs (brain, heart, intestine, kidney, lungs, liver, pancreas, spleen, and stomach) were removed after the rats were euthanized, which was followed by cervical dislocation. The removed organs were preserved in a formalin solution (10% *v*/*v*). All organs were dehydrated for a histological investigation using alcohol submerged in xylene, which was then immersed in paraffin. Furthermore, organ tissues were stained by using hematoxylin and eosin in order to observe pathological changes with light microscopy [22,23,24]. 

#### 2.4.3. Whole Blood Clotting Measurement

Pre-warmed (37 °C) polypropylene tubes were used to hold the pure and thiolated gum ghatti. The dressings were immersed in the desired amount (0.2 mL) of whole blood (citrated) was immersed, and to coagulate the immersed blood, 0.2 M of CaCl_2_ solution (20 µL) was added. The blood in the polypropylene tubes was then incubated for 10 min at 37 °C, with a fixed speed of 30 rpm. Afterwards, free red blood cells were hemolyzed with water (25 mL), and the absorbance value of the resulting hemoglobin solution was analytically measured at 540 nm [25,26,27].

## 3. Results and Discussion

### 3.1. Hematology and Biochemical Test Parameters

After the oral administration of the single doses of pure and thiolated gum ghatti (300 mg/kg body weight) to all Wister rats in each group, they were observed under normal conditions for the first 24 h, and thereafter, they were observed daily for 14 days. Observations revealed no changes to the skin, mucus membrane, or eyes, no hair fall, and circulatory and respiratory conditions, in addition to their behavior patterns were considered normal. Furthermore, there wereno convulsions, tremors, changes in salivation pattern, comas, lethargic states, diarrhea, or changes to sleep pattern seen in groups treated with pure gum or thiolated gum at a concentration of 300 mg/kg and 2000 mg/kg, in comparison to the control group. Moreover, there was no change in body weight during the two weeks of the toxicity study, and no significant difference in food intake. Blood samples of the rats from each group were collected in EDTA vials to perform various clinical test parameters, such as hematological tests (which measure Hb, TLC, DLC, PCV, MCV, MCH, MCHC, Platelet count, and RBC count), liver function tests (which measure total bilirubin, total protein, albumin, SGOT, SGPT, ALP, and total globulin) and renal function tests (which measure blood urea, serum creatinine, serum uric acid, serum potassium, serum sodium, serum calcium, and serum phosphorous). All the results of the aforementioned parameters were found within the normal range, and the statistical data was analyzed using two-way ANOVA followed by Tukey’s post-hoc analysis test. The results of the analysis test showed * *p* < 0.05 vs. control group, as shown in Table 2, and the raw data is available in the supplementary files (Table S1). The study revealed that upon oral administration of pure and thiolated gum ghatti, no toxicity or adverse effects in rats are produced, and thus, they are likely to be safe for potential use as a polymer for the development of oral formulations.

### 3.2. Histopathology Findings

**Figure 3:**
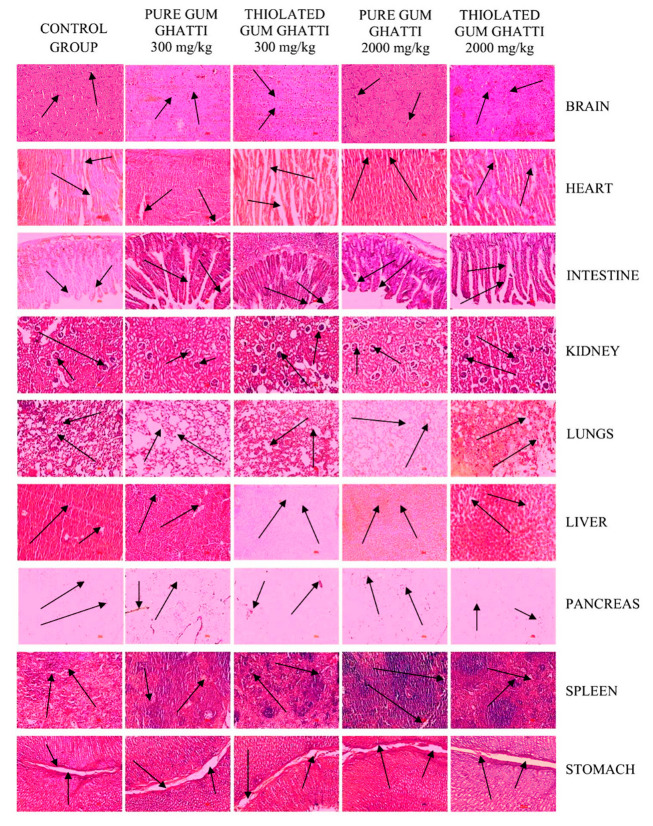
Photomicrographs of the histopathological examination of small, transverse portions of Wistar rat tissues, including the brain, heart, intestine, kidney, lungs, liver, pancreas, spleen, and stomach. Column 1: Control; Column 2: Pure gum ghatti (300 mg/kg body weight); Column 3: Thiolated gum ghatti (300 mg/kg body weight); Column 4: Pure gum ghatti (2000 mg/kg body weight); Column 5: Thiolated gum ghatti (2000 mg/kg body weight).

As per the evidence, no significant histopathological changes were found in the kidney, liver, heart, stomach, intestine, brain, lungs, spleen, or pancreas of the Wistar rats that were treated with pure gum ghatti and thiolated gum ghatti, as depicted in Figure 3. The renal corpuscles were surrounded by the Bowman’s capsule, and the tubules of the kidney were surrounded by the lining of cuboidal epithelial cells. The layers of the heart (i.e.,the endocardium (lines inner surface of heart chambers and valves), epicardium (outer layer of the heart), and myocardium (the muscular tissue)) and the myocardial interstitial tissue were found to be normal. Additionally, in the liver, the structure of the hepatic cell, sinusoidal plate, and hepatic lobule were deemed to be normal. Furthermore, after the oral administration of the synthesized polymer, no ulcerative spots were observed in the stomach. The histopathology of the intestinal sections of the treated Wistar rats were deemed to be as normal as those observed in the control group. In the control and polymer treated slides containing liver cells, hepatic cells were present, thus confirming that the polymer does not induce hepatic cell toxicity. As a result of the above findings, it was shown that the oral administration of single doses of 300 mg/kg and 2000 mg/kg body weight of pure gum ghatti and thiolated gum ghatti, for acute toxicity experiments, had no discernible adverse effects on the vital organs of Wistar rats.

### 3.3. Blood Clotting Factor

**Figure 4:**
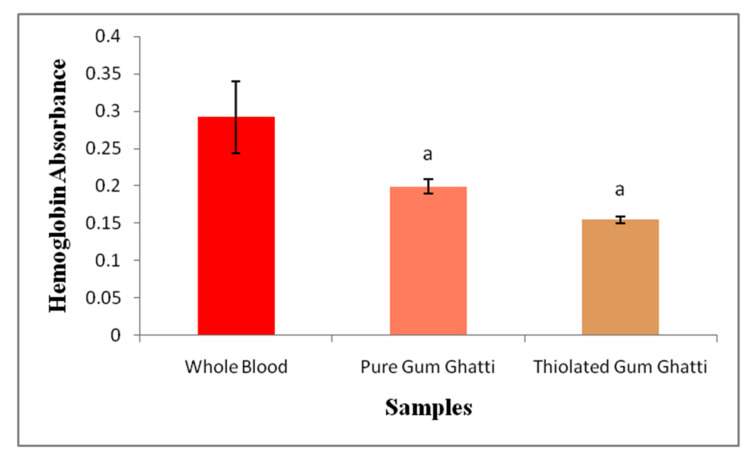
Bar graphic representation of the hemoglobin absorbance of whole blood with respect to pure polymers and thiolated polymers; ‘a’ represents *p* < 0.05 vs. whole blood. Data were analyzed using student’s *t*-Test.

In contrast, to know whether thiolated gum ghatti can increase the blood clotting rate, whole blood needs to make contact with the dressing before hemolyzing the red blood cells for at least 10 min; therefore, the higher the absorbance value, the slower the clotting rate of the hemoglobin solution. Gum ghatti (0.20 nm) and thiolated gum ghatti (0.13 nm) show lower absorbance values than whole blood (0.29 nm), as shown in Figure 4, and the raw data is available in the supplementary files (Table S2). Furthermore, the whole blood clotting factor data were analyzed using the student’s *t*-Test (pure gum ghatti; *p*-value = 0.00259, thiolated gum ghatti; *p*-value = 0.00103 vs. Whole blood). Although there was no statistically significant difference between pure gum ghatti and thiolated gum ghatti (*p*-value > 0.05), both showed significant blood coagulation in contrast to whole blood.

## 4. Conclusions

Thiol modification of gum ghatti has led to the enhancement of mucoadhesive characteristics, and it is implied that this is a promising strategy for the production and development of a sustained release delivery system which targets gastrointestinal, vaginal, ophthalmic, rectal, pulmonary, and buccal drug delivery. Basic characteristics of thiolated biopolymers could be modified for 3D-printed drug delivery systems. The biopolymers’ mucoadhesive and mechanical capabilities were improved by the incorporation of a thiol group into their molecular structure. The successful commercial utilization of this product might be positively investigated, irrespective of the toxicological and regulatory concerns pertaining to the modified biopolymers. The toxicological effects of a single oral dose (300 mg/kg and 2000 mg/kg body weight) of pure gum ghatti and thiolated gum ghatti in an animal (Wistar rat) were investigated, followed by acute oral toxicity studies, which were carried out in compliance with regulatory requirements. After administration, each animal of each individual group was monitored once within the first 30 min, followed by several times within the first 24 h (with a focus on the first 4 h), and once a day thereafter for 14 days. On the 14th day of experiment, blood samples and organs were collected and analyzed for hematological and histopathological studies. The hematology and biochemical test parameters showed that all the results were in the normal range, and no signs of toxicity were observed in the blood of the Wistar rats. Furthermore, histopathological findings revealed no discernible adverse effects on the vital organs of Wistar rats. Blood clotting factors revealed that pure gum ghatti and thiolated gum ghatti had lower absorbance values in contrast to whole blood, which clearly indicates that whole blood and pure gum ghatti, and whole blood and thiolated gum ghatti, experienced the maximum clotting rate in comparison to native whole blood. 

## Figures and Tables

**Table 1:** Treatment schedule for acute oral toxicity studies of pure gum ghatti and thiolated gum ghatti.

Sample No.	Treatment Schedule
1	Group-I (Pure gum ghatti 300 mg/kg, p.o.; *n* = 3)
2	Group-II (Thiolated gum ghatti 300 mg/kg, p.o.; *n* = 3)
3	Group-III (Pure gum ghatti 2000 mg/kg, p.o.; *n* = 3)
4	Group-IV (Thiolated gum ghatti 2000 mg/kg, p.o.; *n* = 3)
5	Group-V (control 0.9% *w*/*v* saline; *n* = 6)

**Table 2:** Single dose comparative analysis of acute oral toxicity showing the effect of gum ghatti and thiolated gum ghatti on hematological parameters, liver profiles, kidney profiles, and feeding behaviors, after testing in experimental Wistar rats.

S. No.	Parameters	Normal Range	Control (0.9% *w*/*v* Saline)	Pure Gum Ghatti (300 mg/kg)	ThiolatedGum Ghatti (300 mg/kg)	Pure Gum Ghatti (2000 mg/kg)	ThiolatedGum Ghatti (2000 mg/kg)
Haematological Test							
1.	Hb (g/dL)	**12.1–15.1**	12.50 ± 0.10	13.20 ± 0.20 *	12.90 ± 0.20 *	13.00 ± 0.17 *	12.83 ± 0.21 *
2.	TLC (Total leucocyte count) (cell/cum)	**4000–11,000**	5877.00 ± 398.14	6105.70 ± 257.99 *	6164.72 ± 181.59 *	6449.57 ± 407.02 *	6217.21 ± 241.35 *
3.	DLC (Differential leucocyte count) (%)						
	Neutrophil	**40–60**	49.53 ± 1.03	49.26 ± 0.84 *	49.92 ± 0.25 *	48.82 ± 0.11 *	49.95 ± 0.16 *
	Lymphocyte	**20–40**	33.00 ± 2.00	35.50 ± 2.10 *	32.20 ± 1.97 *	33.50 ± 1.70 *	33.80 ± 1.42 *
	Monocyte	**2–8**	3.00 ± 0.10	3.20 ± 0.17 *	3.40 ± 0.17 *	3.80 ± 0.17 *	3.20 ± 0.30 *
	Epsinophil	**1–4**	1.90 ± 0.10	2.00 ± 0.26 *	1.80 ± 0.10 *	1.77 ± 0.11 *	2.09 ± 0.18 *
	Basophil	**0.5–1.0**	0.80 ± 0.11	0.90 ± 0.26 *	0.90 ± 0.26 *	1.00 ± 0.17 *	0.90 ± 0.26 *
4.	PCV (packed cell volume) (%)	**35.5–44.9**	43.80 ± 0.10	44.70 ± 0.35 *	44.80 ± 0.10 *	44.20 ± 0.10 *	44.80 ± 0.17 *
5.	MCV (mean corpuscular volume) (fL)	**80–100**	80.80 ± 0.92	78.80 ± 0.26 *	78.70 ± 0.13 *	78.99 ± 0.31 *	79.53 ± 0.59 *
6.	MCH (mean corpuscular hemoglobin) (pg/cell)	**27–31**	29.10 ± 0.20	28.40 ± 0.26 *	28.90 ± 0.10 *	27.10 ± 0.45 *	28.70 ± 0.10 *
7.	MCHC (mean corpuscular hemoglobin concentration) (g/dL)	**32–36**	33.20 ± 1.60	33.00 ± 1.32 *	32.80 ± 1.18 *	33.50 ± 0.90 *	32.90 ± 0.95 *
8.	Platelet count (million/cm^2^)	**150,000–450,000**	1.94 ± 0.06	2.11 ± 0.15 *	1.91 ± 0.06 *	1.99 ± 0.10 *	190 ± 0.08 *
9.	RBC count (million/cm^2^)	**4.2–5.4**	4.40 ± 0.10	4.60 ± 0.20 *	4.50 ± 0.10 *	4.20 ± 0.10 *	4.60 ± 0.10 *
**Liver function test**							
10.	Total bilirubin (mg/dL)	**<1.0–0.4**	0.51 ± 0.06	0.55 ± 0.06 *	0.63 ± 0.06 *	0.59 ± 0.15 *	0.56 ± 0.13 *
11.	Total protein (g/dL)	**6.3–8.0**	6.50 ± 0.20	6.40 ± 0.10 *	6.60 ± 0.20 *	6.50 ± 0.26 *	6.50 ± 0.26 *
12.	Albumin (g/dL)	**3.5–5.5**	4.30 ± 0.10	4.40 ± 0.20 *	4.50 ± 0.20 *	4.60 ± 0.10 *	4.60 ± 0.10 *
13.	SGOT (serum glutamic–oxaloacetic transaminase) (IU/L)	**0–120**	102.00 ± 1.00	118.00 ± 1.00 *	110.00 ± 1.73 *	105.00 ± 2.65 *	105.00 ± 2.65 *
14.	SGPT (serum glutamic–pyruvic transaminase) (IU/L)	**0–60**	43.67 ± 1.15	45.00 ± 0.26 *	40.00 ± 0.95 *	38.00 ± 0.36 *	38.00 ± 0.36 *
15.	ALP (alkaline phosphatase) (IU/L)	**44–147**	110.00 ± 1.30	110.00 ± 1.30 *	140.00 ± 0.78 *	105.31 ± 1.74 *	115.00 ± 0.91 *
16.	Total globulin (g/dL)	**1.5–3.5**	2.20 ± 0.10	2.00 ± 0.08 *	2.10 ± 0.10 *	1.90 ± 0.11 *	1.90 ± 0.11 *
**Kidney function test**							
17.	Blood urea (mg/dL)	**10–50**	32.00 ± 2.00	30.00 ± 1.00 *	30.00 ± 1.00 *	31.00 ± 1.00 *	31.00 ± 1.00 *
18.	Serum creatinine (mg/dL)	**0.6–1.4**	1.00 ± 0.13	1.20 ± 0.20 *	1.10 ± 0.10 *	1.10 ± 0.10 *	1.10 ± 0.10 *
19.	Serum uric acid (mg/dL)	**2.4–7.0**	2.50 ± 0.20	2.00 ± 0.09 *	2.40 ± 0.17 *	2.60 ± 0.10 *	2.60 ± 0.10 *
20.	Serum potassium (mEq/dL)	**3.6–5.5**	4.50 ± 0.10	5.00 ± 0.20 *	4.90 ± 0.10 *	4.40 ± 0.30 *	4.40 ± 0.30 *
21.	Serum sodium (mEq/dL)	**135–155**	148.00 ± 1.85	155.00 ± 0.36 *	155.00 ± 0.36 *	154.71 ± 1.49 *	154.56 ± 0.90 *
22.	Serum calcium (mEq/dL)	**8.5–11.0**	10.00 ± 0.79	9.80 ± 0.10 *	10.20 ± 0.43 *	9.81 ± 0.52 *	9.90 ± 0.35 *
23.	Serum phosphorous (mEq/dL)	**1.5–6.8**	3.80 ± 0.10	3.00 ± 0.26 *	3.20 ± 0.10 *	3.65 ± 0.28 *	3.60 ± 0.26 *
**Physiological and feeding behavior parameters**							
24.	Body weight 0th day (g) 14th day (g)	-	210.20 ± 0.20 210.90 ± 0.30	212.00 ± 0.26 * 212.00 ± 0.20 *	212.20 ± 0.61 * 212.90 ± 0.36 *	211.80 ± 0.17 * 212.50 ± 0.26 *	212.20 ± 0.08 * 212.80 ± 0.10 *
25.	Feed intake 0th day (g/day) 14th day (g/day)	-	12.70 ± 0.17 13.80 ± 0.07	13.21 ± 1.65 * 13.45 ± 0.08 *	13.00 ± 0.20 * 13.90 ± 0.10 *	12.90 ± 0.10 * 13.93 ± 0.58 *	12.70 ± 0.36 * 13.40 ± 0.36 *

## Data Availability

Not applicable.

## Supplementary Materials

The following supporting information can be downloaded at: https://www.mdpi.com/article/10.3390/polym14183836/s1, Figure S1: The SEM technique examined the external morphology of pure gum ghatti and thiolated gum ghatti, which appears to have a rough surface, and the presence of polyhedral flakes in pure gum can also be detected. With regard to the thiolated gum, the image shows a smooth surface with lucent crystalline flakes.; Figure S2: X-ray diffractogram of pure gum ghatti indicates an amorphous nature with no sharp peak, where as thiolated gum ghatti shows sharp peaks at 42.32, and 50.85, (2θ), suggesting substantial crystalline activity.; Figure S3: FTIR spectrum of pure gum ghatti and thiolated gum ghatti. FTIR spectrum of pure gum ghatti shows characteristic peaks at 3418.78 cm^−1^ (–OH stretch), 2932.28 cm^−1^ (–CH stretch), 2131.47 cm^−1^ (C≡C stretch), and 1639.44 cm^−1^ (C=C alkene stretch). In the FTIR spectrum of thiolated gum ghatti, it shows additional peaks at 2567.39 cm^−1^ (–SH stretch) and 1720.91 cm^−1^(C=O ester stretch), which confirms the thiolation of gum ghatti.; Table S1: Raw data showing a single dose comparative analysis of acute oral toxicity which demonstrates the effect of gum ghatti and thiolated gum ghatti on the hematological parameters, liver profiles, kidney profiles, and feeding behaviors after testing was conducted upon experimental Wistar rats.; Table S2: Mean and Standard Deviation values of the Blood Clotting Factor.
