# Supplementary material for: Assessment of Acute Oral Toxicity of Thiolated Gum Ghatti in Rats
Source: Polymers (Basel). 2022 Sep 14;14(18):3836. doi: 10.3390/polym14183836 (PMC9506416; doi:10.3390/polym14183836)
Supplement: Supplementary file 1 [file polymers-14-03836-s001.zip › polymers-1892511-supplementary.pdf]

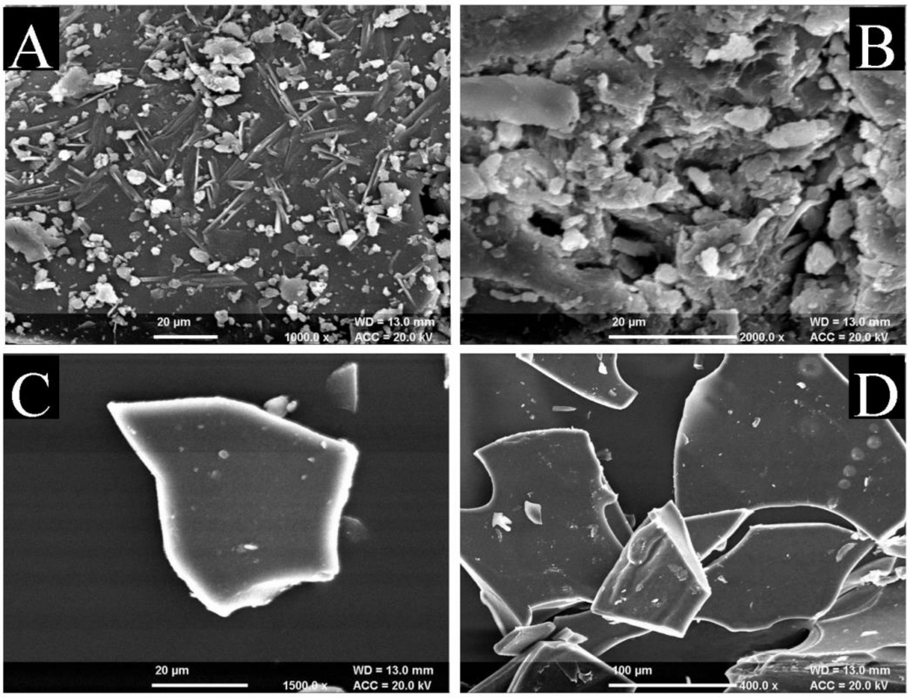

Figure S1. The SEM technique examined the external morphology of pure gum ghatti and thiolated gum ghatti which appears to be rough surface and with presence of polyhedral flakes in pure gum; and in thiolated gum image shows smooth surface with lucent crystalline flakes.

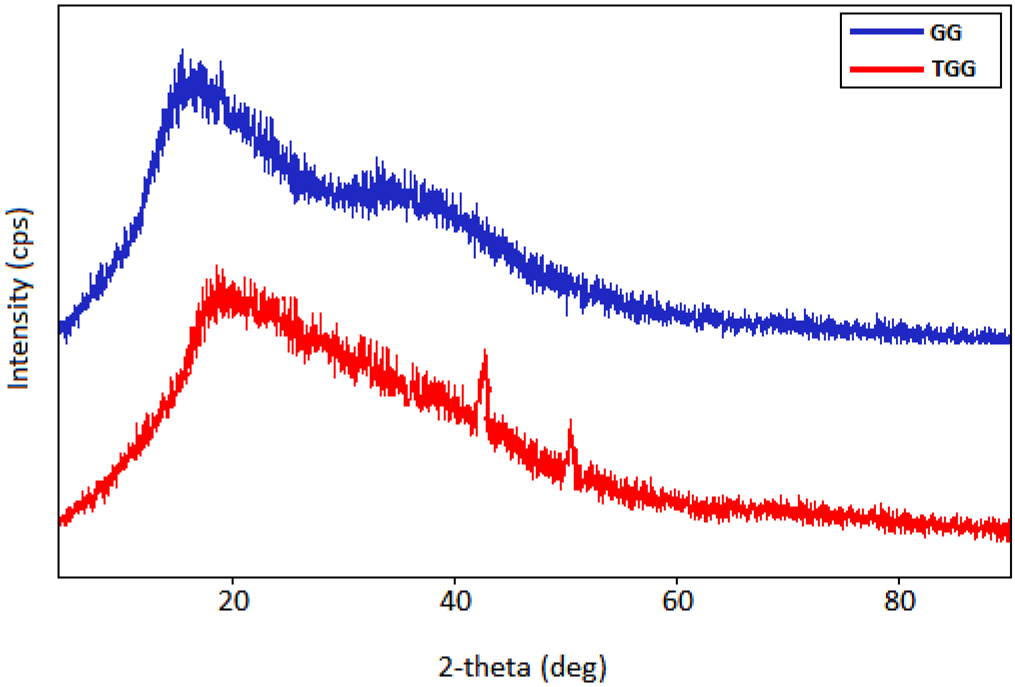

Figure S2. X-ray diffractogram of pure gum ghatti indicates amorphous nature with no sharp peak, while thiolated gum ghatti shows sharp peaks at 42.32, and 50.85, (2ϴ), suggesting substantial crystalline activity.

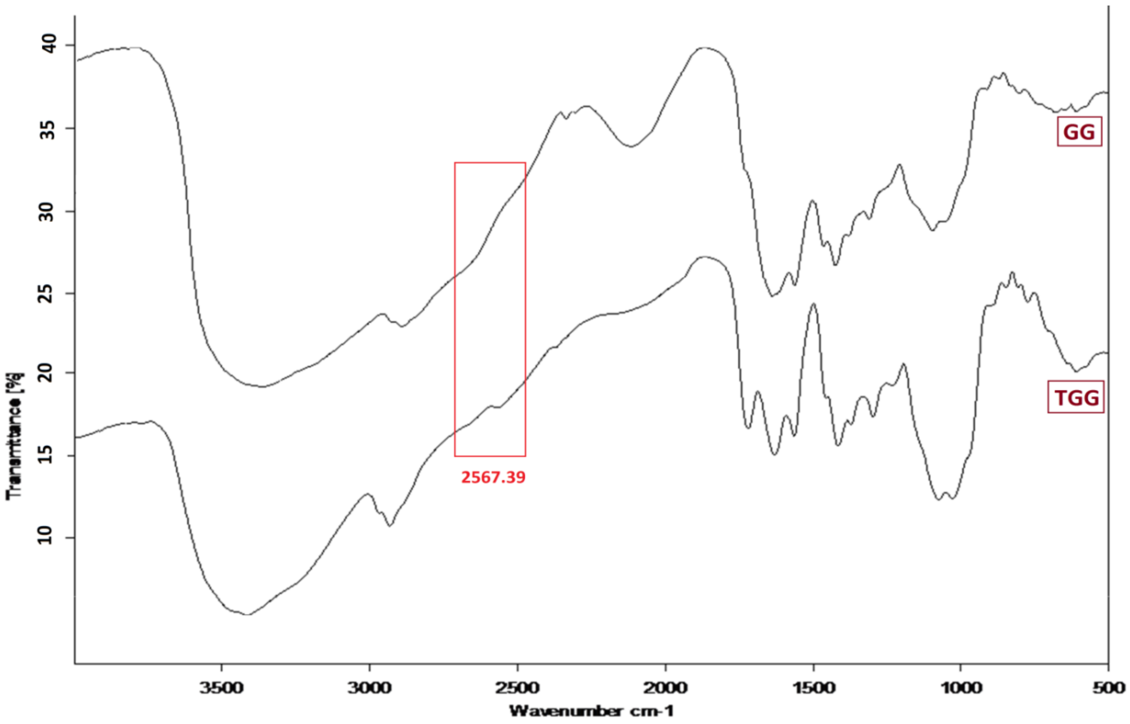

Figure S3. FTIR spectrum of pure gum ghatti and thiolated gum ghatti. FTIR spectrum of pure gum ghatti shows characteristics peaks at 3418.78 cm^{-1} (–OH stretch), 2932.28 cm^{-1} (-CH stretch), 2131.47 cm^{-1} (C≡C stretch), and 1639.44 cm^{-1} (C=C alkene stretch). In the FTIR spectrum of thiolated gum ghatti shows additional peaks at 2567.39 cm^{-1} (-SH stretch) and 1720.91 cm^{-1} (C=O ester stretch) confirms the thiolation of gum ghatti.

Table S1. Raw data of single dose comparative analysis of acute oral toxicity showing the effect of gum ghatti and thiolated gum ghatti on haematological parameters, liver profile, kidney profile and feeding behavioural test in experimental Wistar rats

S. No.	Parameters	Control Sample					Pure gum ghatti (300 mg/kg)					Thiolated gum ghatti (300 mg/kg)					Pure gum ghatti (2000 mg/kg)					Thiolated gum ghatti (2000 mg/kg)				
		1.00	2.00	3.00	Avg	SD	1.00	2.00	3.00	Avg	SD	1.00	2.00	3.00	Avg	SD	1.00	2.00	3.00	Avg	SD	1.00	2.00	3.00	Avg	SD
1	Hb (g/dL)	12.40	12.60	12.50	**12.50**	**0.10**	13.00	13.20	13.40	**13.20**	**0.20**	12.70	12.90	13.10	**12.90**	**0.20**	12.90	12.90	13.20	**13.00**	**0.17**	12.60	12.90	13.00	**12.83**	**0.21**
2	TLC (Total leucocyte count) (cell/cum)	5840.54	6292.12	5498.34	**5877.00**	**398.14**	5892.12	6392.34	6032.65	**6105.70**	**257.99**	6368.97	6021.54	6103.65	**6164.72**	**181.59**	6765.76	5990.34	6592.61	**6449.57**	**407.02**	5976.62	6459.31	6215.71	**6217.21**	**241.35**
3	Neutrophil	50.12	48.34	50.12	**49.53**	**1.03**	48.80	50.23	48.75	**49.26**	**0.84**	50.20	49.70	49.87	**49.92**	**0.25**	48.70	48.92	48.84	**48.82**	**0.11**	49.85	49.87	50.14	**49.95**	**0.16**
4	Lymphocyte	31.00	33.00	35.00	**33.00**	**2.00**	33.40	37.60	35.50	**35.50**	**2.10**	30.28	32.11	34.21	**32.20**	**1.97**	32.20	32.87	35.43	**33.50**	**1.70**	33.10	32.87	35.43	**33.80**	**1.42**
5	Monocyte	2.90	3.10	3.00	**3.00**	**0.10**	3.10	3.40	3.10	**3.20**	**0.17**	3.30	3.60	3.30	**3.40**	**0.17**	3.70	3.70	4.00	**3.80**	**0.17**	3.20	2.90	3.50	**3.20**	**0.30**
6	Epsinophil	1.80	2.00	1.90	**1.90**	**0.10**	1.80	2.30	1.90	**2.00**	**0.26**	1.80	1.70	1.90	**1.80**	**0.10**	1.68	1.89	1.75	**1.77**	**0.11**	2.25	2.12	1.90	**2.09**	**0.18**
7	Basophil	0.70	0.92	0.78	**0.80**	**0.11**	0.70	1.20	0.80	**0.90**	**0.26**	0.70	1.20	0.80	**0.90**	**0.26**	0.90	1.20	0.90	**1.00**	**0.17**	0.80	0.70	1.20	**0.90**	**0.26**
8	PCV (packed cell volume) (%)	43.70	43.90	43.80	**43.80**	**0.10**	44.90	44.30	44.90	**44.70**	**0.35**	44.70	44.80	44.90	**44.80**	**0.10**	44.30	44.20	44.10	**44.20**	**0.10**	44.90	44.60	44.90	**44.80**	**0.17**
9	MCV (mean corpuscular volume) (fL)	80.24	81.87	80.30	**80.80**	**0.92**	78.60	78.70	79.10	**78.80**	**0.26**	78.56	78.75	78.80	**78.70**	**0.13**	79.34	78.87	78.75	**78.99**	**0.31**	80.22	79.20	79.18	**79.53**	**0.59**
10	MCH (mean corpuscular hemoglobin) (pg/cell)	29.09	29.30	28.90	**29.10**	**0.20**	28.30	28.70	28.20	**28.40**	**0.26**	28.80	28.91	29.00	**28.90**	**0.10**	26.98	26.72	27.60	**27.10**	**0.45**	28.80	28.70	28.60	**28.70**	**0.10**
11	MCHC (mean corpuscular haemoglobin concentration) (g/dL)	32.50	35.03	32.07	**33.20**	**1.60**	32.50	34.50	32.00	**33.00**	**1.32**	32.50	34.10	31.80	**32.80**	**1.18**	33.50	34.40	32.60	**33.50**	**0.90**	33.50	33.40	31.80	**32.90**	**0.95**
12	Platelet count (million/cm^2)	1.88	1.94	1.99	**1.94**	**0.06**	1.94	2.17	2.23	**2.11**	**0.15**	1.96	1.92	1.85	**1.91**	**0.06**	2.10	1.94	1.93	**1.99**	**0.10**	1.95	1.94	1.80	**1.90**	**0.08**
13	RBC count (million/cm^2)	4.40	4.30	4.50	**4.40**	**0.10**	4.40	4.60	4.80	**4.60**	**0.20**	4.40	4.60	4.50	**4.50**	**0.10**	4.20	4.30	4.10	**4.20**	**0.10**	4.60	4.70	4.50	**4.60**	**0.10**
14	Total bilirubin (mg/dL)	0.47	0.49	0.58	**0.51**	**0.06**	0.52	0.51	0.61	**0.55**	**0.06**	0.60	0.60	0.70	**0.63**	**0.06**	0.43	0.72	0.61	**0.59**	**0.15**	0.43	0.68	0.58	**0.56**	**0.13**
15	Total protein (g/dL)	6.70	6.30	6.50	**6.50**	**0.20**	6.50	6.30	6.40	**6.40**	**0.10**	6.80	6.60	6.40	**6.60**	**0.20**	6.80	6.30	6.40	**6.50**	**0.26**	6.80	6.30	6.40	**6.50**	**0.26**
16	Albumin (g/dL)	4.20	4.40	4.30	**4.30**	**0.10**	4.60	4.40	4.20	**4.40**	**0.20**	4.50	4.30	4.70	**4.50**	**0.20**	4.50	4.70	4.60	**4.60**	**0.10**	4.50	4.70	4.60	**4.60**	**0.10**
17	SGOT (serum glutamic-oxaloacetic transaminase) (IU/L)	101.00	103.00	102.00	**102.00**	**1.00**	118.00	119.00	117.00	**118.00**	**1.00**	109.00	109.00	112.00	**110.00**	**1.73**	103.00	104.00	108.00	**105.00**	**2.65**	103.00	104.00	108.00	**105.00**	**2.65**
18	SGPT (serum glutamic-pyruvic transaminase) (IU/L)	43.00	43.00	45.00	**43.67**	**1.15**	45.30	44.90	44.80	**45.00**	**0.26**	40.09	40.90	39.01	**40.00**	**0.95**	37.90	37.70	38.40	**38.00**	**0.36**	37.90	37.70	38.40	**38.00**	**0.36**
19	ALP (alkaline phosphatase) (IU/L)	111.50	109.20	109.30	**110.00**	**1.30**	111.50	109.20	109.30	**110.00**	**1.30**	139.18	140.08	140.74	**140.00**	**0.78**	105.20	107.10	103.63	**105.31**	**1.74**	115.96	114.15	114.90	**115.00**	**0.91**
20	Total globulin (g/dL)	2.30	2.10	2.20	**2.20**	**0.10**	2.10	1.96	1.95	**2.00**	**0.08**	2.10	2.00	2.20	**2.10**	**0.10**	2.01	1.80	1.90	**1.90**	**0.11**	2.01	1.80	1.90	**1.90**	**0.11**
21	Blood urea (mg/dL)	30.00	32.00	34.00	**32.00**	**2.00**	30.00	29.00	31.00	**30.00**	**1.00**	30.00	29.00	31.00	**30.00**	**1.00**	31.00	30.00	32.00	**31.00**	**1.00**	31.00	30.00	32.00	**31.00**	**1.00**
22	Serum creatinine (mg/dL)	0.85	1.09	1.06	**1.00**	**0.13**	1.00	1.40	1.20	**1.20**	**0.20**	1.00	1.10	1.20	**1.10**	**0.10**	1.00	1.10	1.20	**1.10**	**0.10**	1.00	1.10	1.20	**1.10**	**0.10**
23	Serum uric acid (mg/dL)	2.70	2.50	2.30	**2.50**	**0.20**	1.94	1.97	2.10	**2.00**	**0.09**	2.50	2.50	2.20	**2.40**	**0.17**	2.50	2.60	2.70	**2.60**	**0.10**	2.50	2.60	2.70	**2.60**	**0.10**
24	Serum potassium (mEq/dL)	4.40	4.60	4.50	**4.50**	**0.10**	5.20	4.80	5.00	**5.00**	**0.20**	4.90	4.80	5.00	**4.90**	**0.10**	4.10	4.40	4.70	**4.40**	**0.30**	4.10	4.40	4.70	**4.40**	**0.30**
25	Serum sodium (mEq/dL)	148.10	149.80	146.10	**148.00**	**1.85**	155.10	155.30	154.60	**155.00**	**0.36**	155.10	155.30	154.60	**155.00**	**0.36**	153.14	154.87	156.11	**154.71**	**1.49**	154.97	153.53	155.19	**154.56**	**0.90**
26	Serum calcium (mEq/dL)	9.09	10.40	10.50	**10.00**	**0.79**	9.70	9.90	9.80	**9.80**	**0.10**	9.70	10.39	10.50	**10.20**	**0.43**	10.11	10.10	9.21	**9.81**	**0.52**	10.11	10.10	9.50	**9.90**	**0.35**
27	Serum phosphorous (mEq/dL)	3.90	3.70	3.80	**3.80**	**0.10**	2.80	3.30	2.90	**3.00**	**0.26**	3.10	3.30	3.20	**3.20**	**0.10**	3.45	3.53	3.97	**3.65**	**0.28**	3.40	3.50	3.90	**3.60**	**0.26**
28	Body weight 0th day (g)	210.20	210.40	210.00	**210.20**	**0.20**	212.30	211.90	211.80	**212.00**	**0.26**	212.90	211.90	211.80	**212.20**	**0.61**	211.60	211.90	211.90	**211.80**	**0.17**	212.30	212.15	212.16	**212.20**	**0.08**
29	14th day (g)	210.60	210.90	211.20	**210.90**	**0.30**	212.00	212.20	211.80	**212.00**	**0.20**	213.30	212.60	212.80	**212.90**	**0.36**	212.70	212.20	212.60	**212.50**	**0.26**	212.90	212.80	212.70	**212.80**	**0.10**
30	Feed intake 0th day (g/day)	12.50	12.80	12.80	**12.70**	**0.17**	14.45	13.83	11.34	**13.21**	**1.65**	12.80	13.00	13.20	**13.00**	**0.20**	12.80	12.90	13.00	**12.90**	**0.10**	12.80	12.30	13.00	**12.70**	**0.36**
31	14th day (g/day)	13.76	13.75	13.88	**13.80**	**0.07**	13.54	13.40	13.42	**13.45**	**0.08**	13.80	14.00	13.90	**13.90**	**0.10**	14.56	13.43	13.80	**13.93**	**0.58**	13.80	13.30	13.10	**13.40**	**0.36**

Table S2. Mean and Standard Deviation values of Blood Clotting Factor

Sample	Mean	Std. Dev.
Whole Blood	0.292	0.048682
Pure Gum Ghatti	0.199	0.009555
Thiolated Gum Ghatti	0.155	0.004403
